# Association between Mouth Breathing and Atopic Dermatitis in Japanese Children 2–6 years Old: A Population-Based Cross-Sectional Study

**DOI:** 10.1371/journal.pone.0125916

**Published:** 2015-04-27

**Authors:** Harutaka Yamaguchi, Saaya Tada, Yoshinori Nakanishi, Shingo Kawaminami, Teruki Shin, Ryo Tabata, Shino Yuasa, Nobuhiko Shimizu, Mitsuhiro Kohno, Atsushi Tsuchiya, Kenji Tani

**Affiliations:** 1 Department of General Medicine, Institute of Health Biosciences, The University of Tokushima Graduate School, Tokushima City, Japan; 2 Tokushima Prefecture Naruto Hospital, Naruto City, Japan; 3 Outpatient Facility Kameda Clinic, Tateyama City, Japan; 4 Department of Civil and Environmental Studies, Faculty of Integrated Arts and Sciences, The University of Tokushima Graduate School, Tokushima City, Japan; Örebro University, SWEDEN

## Abstract

As mouth breathing is associated with asthma and otitis media, it may be associated with other diseases. Therefore, this population-based cross-sectional study evaluated the association of mouth breathing with the prevalences of various diseases in children. Preschool children older than 2 years were included. A questionnaire was given to parents/guardians at 13 nurseries in Tokushima City. There were 468 valid responses (45.2%). We defined a subject as a mouth breather in daytime (MBD) if they had 2 or more positive items among the 3 following items: “breathes with mouth ordinarily,” “mouth is open ordinarily,” and “mouth is open when chewing.” We defined subjects as mouth breathers during sleep (MBS) if they had 2 or more positive items among the following 3 items: “snoring,” “mouth is open during sleeping,” and “mouth is dry when your child gets up.” The prevalences of MBD and MBS were 35.5% and 45.9%, respectively. There were significant associations between MBD and atopic dermatitis (odds ratio [OR]: 2.4, 95% confidence interval [CI]: 1.4–4.2), MBS and atopic dermatitis (OR: 2.4, 95% CI: 1.3–4.2), and MBD and asthma (OR: 2.2, 95% CI: 1.2–4.0). After adjusting for history of asthma and allergic rhinitis; family history of atopic dermatitis, asthma, and allergic rhinitis; and nasal congestion; both MBD (OR: 2.6, 95% CI: 1.3–5.4) and MBS (OR: 4.1, 95% CI: 1.8–9.2) were significantly associated with atopic dermatitis. In preschool children older than 2 years, both MBD and MBS may be associated with the onset or development of atopic dermatitis.

## Introduction

The prevalence of mouth breathing among children remains controversial but is at most reported to be 50–56% [1–8]. Mouth breathing is defined as using the mouth alone or the mouth and nose instead of the nose alone for respiration for longer than 6 months [9]. Mouth breathing is thought to be caused by mechanical factors such as septal deviation and adenotonsillar hyperplasia, inflammatory diseases such as allergic rhinitis, congenital malformation, and behavioral mouth breathing [9, 10].

The functions of the nasal cavity are air-conditioning, olfaction, and defense [11], but mouth breathing causes environmental air to bypass these nasal functions, allowing air to directly enter the lower respiratory tract, which can cause airway hyperreactivity and chronic bronchial inflammation [12–14]. Two case–control studies showed that children suffering from asthma exhibit more mouth breathing behaviors than controls [15, 16]. Meanwhile, a cohort study revealed that the risk of otitis media with effusion is 2.4 times higher in mouth breathers than nose breathers [17].

In addition, mouth breathing might be associated with skin diseases, given its previously demonstrated relationships with periodontal disease and enlarged tonsils. Periodontal disease is associated with chronic skin diseases such as chronic urticaria [18], chronic pigmented purpura [19], and chronic nodular prurigo [20]. Mouth breathers had an increased risk of gingivitis in a case-control study [21], while patients suffering from adenotonsillar hypertrophy, which is a cause of mouth breathing, have an increased risk of periodontal disease, which improves after adenoidectomy [22]. In addition, Valera et al. reported that children aged 3–6 years with enlarged tonsils had a significantly increased risk of mouth breathing [23]. Streptococcal tonsillitis is associated with psoriasis [24], while some reports indicate tonsillectomy improves psoriasis [25, 26].

However, no population-based studies have investigated the relationship between mouth breathing and the prevalences of pediatric diseases, including atopic dermatitis, which is a highly prevalent skin disease in children [27].

Accordingly, this study investigated the relationship of mouth breathing with the prevalences of various diseases including atopic dermatitis by using a questionnaire targeting preschool children in day nurseries.

## Materials and Methods

### Design, setting, and participants

Aimed to have a total of 600 respondents, we targeted preschool children aged 2–6 years who attended day nurseries in Tokushima City. The questionnaire was distributed at 13 randomly selected day nurseries in Tokushima City. We distributed anonymous questionnaires to the parents or guardians from November 27 to December 16, 2013. The questionnaires were submitted through collection boxes in each day nursery.

This was a population-based cross-sectional study performed with the permission of the Ethics Committee of the Tokushima University Hospital. After obtaining written informed consent from the head of each day nursery, we distributed the questionnaires to parents/guardians attached with an explanatory leaflet specifying that their submission of the questionnaire was considered consent. Within the leaflet, we also informed the parents/guardians that some, as yet unknown, behavioral habits might contribute to the development of some diseases; however, we did not provide information regarding the specific potential associations.

### Questionnaire

The questionnaire included questions on the following: age, sex, smoking habits of family members, behavioral habits, present and previous diseases, and present and previous diseases of parents. A multiple choice question was used to collect information about present diseases, including allergic rhinitis, chronic sinusitis, asthma, chronic bronchitis, pollinosis, atopic dermatitis, tonsillitis, otitis media, chronic headache, proteinuria, and hematuria. In addition to the present diseases, previous diseases included acute sinusitis, acute otitis media, chronic otitis media, pneumonia, and meningitis. As some cases of allergic rhinitis are pollinosis [28], we treated pollinosis as allergic rhinitis. Excluding mouth breathing, which is described in the next section, we collected information about the following behavioral habits: regular bedtime and rising time, sleeping hours, sleeping posture, pacifier use, and dietary habits including mastication and food and drink preferences.

### Mouth breathing criteria

Although there is no widely adopted questionnaire for evaluating mouth breathing, we prepared the following 3 items for detecting mouth breathers in daytime (MBD) (Table 1): “breathes with mouth ordinarily,” “mouth is open ordinarily,” and “mouth is open when chewing.” These items were developed with reference to generally used methods including the Glatzel mirror, lip closure, and the water test [29]. “Breathes with mouth ordinarily” corresponds to the Glatzel mirror, which judges mouth breathing by vapor emanating from the mouth using a mirror placed below the child’s nose. “Mouth is open ordinarily” corresponds to lip closure, which is determined according to soft contact between the upper and lower lips. “Mouth is open when chewing” corresponds to the water test, in which children hold some water in their mouth while keeping their lips closed without swallowing for 3 minutes. The necessity of opening the mouth during mastication suggests the child is not in the habit of complete nose breathing. Children who met 0–1 and 2–3 of the above criteria were considered nasal breathers in daytime (NBD) and MBD, respectively.

**Table 1:** Questionnaires for mouth breathing.

Questions	Choices			
**Items for MBD**				
Breathes with mouth ordinarily	Nose usually	Mouth usually	Mouth	Nose and mouth
Mouth is open ordinarily	Usually closed	Sometimes open	Often open	Always open
Mouth is open when chewing	Usually closed	Usually open	Both are applicable	
**Items for MBS**				
Snoring	Not at all	Not usually	Sometimes	Often
Mouth is open during sleep	Not at all	Not usually	Sometimes	Often
Mouth is dry when your child gets up	Wet	A little dry	Very dry	

Underlined and non-underlined choices were considered positive and negative for mouth breathing, respectively. MBD: mouth breather in daytime, MBS: mouth breather during sleep.

We adopted the following 3 items to detect mouth breathers during sleep (MBS): “snoring,” “mouth is open during sleep,” and “mouth is dry when your child gets up.” Snoring is significantly associated with MBD and MBS [5, 30]. Dry mouth is caused by dry air passing through the mouth unless a loss of saliva occurs [31]. Children who met 0–1 and 2–3 criteria were considered nasal breathers during sleep (NBS) and MBS, respectively.

For each item, we defined positive or negative choices for mouth breathing before distributing the questionnaire. As shown in Table 1, the underlined and non-underlined choices were defined as positive and negative for mouth breathing, respectively. We did not indicate in the questionnaire which questions were used to analyze mouth breathing or which choices were used to determine mouth breathing.

Complete mouth breathers (CMB) were defined as children who met the criteria for both MBD and MBS, partial mouth breathers (PMB) met the criteria for either MBD or MBS, and complete nasal breathers (CNB) met the criteria for both NBD and NBS.

Because nasal congestion, which is the main symptom of rhinitis and sinusitis, is an important cause of mouth breathing, we included an item for “nasal congestion”; it was assessed as negative only if “not blocked unless cold” was selected and assessed as positive if “often blocked” or “always blocked” was selected.

Genetic factors were considered positive if the parents selected the corresponding disease from among present and/or previous diseases, because atopic dermatitis and asthma improve naturally with age and allergic rhinitis is classified as intermittent, seasonal, or persistent according to the nature of the allergen. Smoking was categorized according to whether family member(s) smoked around the children or not.

### Statistical analysis

Statistical analyses were performed by using SPSS version 21 (IBM Corp, Armonk, NY, USA). Continuous variables are presented as mean ± standard deviation (SD) or median and interquartile range (IQR).

For univariate analysis, categorical variables such as habits and disease presence were analyzed by Pearson’s *χ* ^2^ tests or Fisher’s exact tests where appropriate. The results are presented as odds ratios (ORs) and 95% confidence intervals (CIs). To adjust the ORs of MBD and MBS with respect to disease prevalence, the Mantel–Haenszel test was performed using variables with *p* < 0.25 in univariate analysis as potential confounders.

For multivariate analysis, forward stepwise multiple logistic regression was performed by using breathing pattern (i.e., MBD or MBS) as the dependent variable and categorical variables showing *p* < 0.25 in univariate analysis as independent variables. Bonferroni corrections for multiple comparisons were made as a *post hoc* analysis. The level of significance was set at *p* < 0.05 except in cases of multiple comparisons, in which *p* < 0.016 was used.

We used the phi coefficient to indicate the effect size in the Pearson’s *χ* ^2^ test. For statistical power, we performed *post hoc* power analysis using G*power (ver. 3.1.9.2, Erdfelder, Faul and Buchuner, Germany) [32].

## Results

We distributed questionnaires to 1036 subjects from November 27 to December 16, 2013 and collected 552 responses for a response rate of 53.3%. No reminder letters were distributed. Thirteen responses were excluded because of age: 11 were outside the target age range and 2 had no age given. A further 71 responses were excluded because of at least one unanswered question. Therefore, a total of 468 valid responses were collected for a response rate of 45.2%.

The subjects’ characteristics are shown in Table 2. Mean age was 4.5 ± 1.2 years with a median of 4.5 years (IQR: 3.4–5.5 years). The subjects were divided into 3 groups according to the main diseases reported: atopic dermatitis, asthma, and allergic rhinitis. Children with atopic dermatitis were significantly associated with a history of asthma and/or allergic rhinitis. In contrast, asthmatic children were significantly associated with a history of allergic rhinitis, atopic dermatitis, and/or pneumonia. The most afflicted children were those with allergic rhinitis, who had a history of all documented conditions except tonsillitis. Each group had a family history of their condition, and the atopic dermatitis group was also significantly associated with a family history of allergic rhinitis.

**Table 2:** Characteristics of total subjects and subjects by disease.

	Total	Atopic dermatitis	Asthma	Allergic rhinitis
	*n* (%)	*n* (%)	*n* (%)	*n* (%)
**Age** (years)				^#^
2	62 (13.2)	9 (14.5)	8 (12.9)	5 (8.1)
3	117 (25.0)	13 (11.1)	8 (6.8))	12 (10.3)
4	111 (23.7)	10 (9.0)	14 (12.6)	14 (12.6)
5	106 (22.6)	17 (16.0)	10 (9.4)	15 (14.2)
6	72 (15.4)	10 (13.9)	6 (8.3)	15 (20.8)
Total	468 (100.0)	59 (12.6)	46 (9.8)	61 (13.0)
**Sex**				
Male	253 (54.1)	33 (55.9)	28 (60.9)	45 (73.8)***
**History**				
Atopic dermatitis	72 (15.4)	-	15 (32.6)***	23 (37.7)***
Asthma	68 (14.5)	20 (33.9)***	-	17 (27.9)**
Allergic rhinitis	85 (18.2)	25 (42.4)***	15 (32.6)**	-
Pneumonia	55 (11.8)	6 (10.2)	10 (21.7)*	12 (19.7)*
Tonsillitis	59 (12.6)	6 (10.2)	5 (10.9)	11 (18.0)^#^
Chronic otitis media	36 (7.7)	4 (6.8)	4 (8.7)	10 (16.4)* ^$^
**Family history**				
Atopic dermatitis	102 (21.8)	36 (61.0)***	9 (19.6)	15 (24.6)
Asthma	81 (17.3)	14 (23.7)^#^	21 (45.7)***	14 (23.0)^#^
Allergic rhinitis	312 (66.7)	47 (79.7)*	33 (71.7)	57 (93.4)***
**Smoking**				
Smokes around children	33 (7.1)	4 (6.8)	4 (8.7)	1 (1.6)^#^ ^$^
Any smoker	215 (45.9)	24 (40.7)	21 (45.7)	26 (42.6)

History of atopic dermatitis, asthma, and allergic rhinitis included both present and previous disease. *P*-values were calculated using Pearson’s *χ* ^2^ test.

^$^: Fisher’s exact test.

^#^ *p* < 0.25,

* *p* < 0.05,

** *p* < 0.01,

*** *p* < 0.001

The results show that 57.5% of the children had substantial nasal breathing difficulties, including CMB (23.9%) and PMB (33.5%). The symptoms appeared to be more frequent at night; 45.9% were MBS compared to 35.5% who were MBD (Table 3).

**Table 3:** Association between mouth breathing and disease prevalence.

		Total	Atopic dermatitis				Asthma				Allergic rhinitis			
			-	+			-	+			-	+		
		*n* = 468 (%)	*n* = 409 (%)	*n* = 59 (%)	OR	95% CI	*n* = 422 (%)	*n* = 46 (%)	OR	95% CI	*n* = 407 (%)	*n* = 61 (%)	OR	95% CI
**MBD**	Positive	166	134	32	2.43	1.40–4.23**	142	24	2.15	1.17–3.97*	137	29	1.79	1.04–3.07*
		(35.5)	(80.7)	(19.3)			(85.5)	(14.5)			(82.5)	(17.5)		
	Negative	302	275	27			280	22			270	32		
		(64.5)	(91.1)	(8.9)			(92.7)	(7.3)			(89.4)	(10.6)		
**MBS**	Positive	215	177	38	2.37	1.34–4.18**	192	23	1.20	0.65–2.20	177	38	2.15	1.23–3.73**
		(45.9)	(82.3)	(17.7)			(89.3)	(10.7)			(82.3)	(17.7)		
	Negative	253	232	21			230	23			230	23		
		(54.1)	(91.7)	(8.3)			(90.9)	(9.1)			(90.9)	(9.1)		

*P*-values were calculated using Pearson’s *χ* ^2^ test. OR: odds ratio, CI: confidence interval, MBD: mouth breather in daytime, MBS: mouth breather during sleep.

* *p* < 0.05,

** *p* < 0.01

Atopic dermatitis was significantly associated with both MBD and MBS (*p* = 0.001 and *p* = 0.002, respectively). Asthma was significantly associated with only MBD (*p* = 0.013), while allergic rhinitis was significantly associated with both MBD and MBS (*p* = 0.035 and 0.006, respectively). Nasal congestion was significantly associated with a risk of all 3 diseases, which was considered a confounder (S1 Appendix). Also, nasal congestion, which was present with [4/97 (4.1%)] and without [1/371 (0.3%)] chronic sinusitis, was associated with chronic sinusitis (OR: 15.9, 95% CI: 1.8–144.1, *p* = 0.007, Fisher’s exact test).

According to the results of the univariate analysis, we adopted previous disease (i.e., asthma and allergic rhinitis), family history of disease (i.e., atopic dermatitis, asthma, and allergic rhinitis), and nasal congestion as confounding factors for atopic dermatitis. Meanwhile, previous disease (i.e., atopic dermatitis, allergic rhinitis, and pneumonia), family history of disease (i.e., asthma), and nasal congestion were adopted as confounding factors for asthma. Because nasal congestion can induce mouth breathing and is one of the main symptoms of allergic rhinitis, we excluded allergic rhinitis from subsequent analyses. After adjusting for confounders, atopic dermatitis was significantly associated with risks of both MBD (OR: 2.6, 95% CI: 1.3–5.4, *p* = 0.010) and MBS (OR: 4.1, 95% CI: 1.8–9.2, *p* = 0.001), although asthma was not significantly associated with a risk of MBD (OR: 1.3, 95% CI: 0.7–2.7, *p* = 0.508). Multiple logistic regression for atopic dermatitis was subsequently performed by including the categorical variables listed above. The ORs of MBD and MBS for atopic dermatitis were 2.2 (95% CI: 1.2–4.2) and 2.7 (95% CI: 1.4–5.3), respectively (Table 4); these values were lower than those for family history of atopic dermatitis and history of asthma and allergic rhinitis. Nasal congestion and parental history of asthma and allergic rhinitis were not significant risk factors.

**Table 4:** Multiple logistic regression analysis of factors associated with atopic dermatitis.

	MBD			MBS		
	OR	95% CI		OR	95% CI	
Mouth breathing	2.19	1.15–4.15	*	2.71	1.40–5.25	**
History of allergic rhinitis	4.67	2.31–9.43	***	4.69	2.32–9.49	***
History of asthma	4.71	2.23–9.98	***	5.15	2.42–10.99	***
Parental history of atopic dermatitis	12.51	6.24–25.05	***	13.56	6.69–27.47	***

Breathing pattern (i.e., MBD or MBS) was the dependent variable, and the following variables showing *p* < 0.25 in the univariate analysis were included as independent variables: previous disease (i.e., asthma and allergic rhinitis), parental disease history (i.e., atopic dermatitis, asthma, and allergic rhinitis), and nasal congestion (*n* = 468). MBD: mouth breather in daytime, MBS: mouth breather during sleep, OR: odds ratio, 95% CI: 95% confidence interval.

* *p* < 0.05,

** *p* < 0.01,

***p < 0.001.

The effect sizes of MBD and MBS for atopic dermatitis were 0.149 and 0.141, respectively, and 0.897 and 0.862, respectively, represented the statistical power in the *post hoc* analysis, indicating sufficient power.

Atopic dermatitis was present in 7.0% of children with CNB (n = 199), 12.7% of children with PMB (n = 157), and 22.3% of children with CMB (n = 112) (*p* < 0.001). The prevalence of atopic dermatitis in CMBs was higher than that in PMBs (*p* = 0.038), but the difference was not significant after Bonferroni correction.

## Discussion

The results of the present study indicate mouth breathing is significantly associated with atopic dermatitis and asthma. In particular, atopic dermatitis was significantly associated with both MBD and MBS after adjusting for confounding factors. Moreover, the prevalences of these diseases showed an increasing trend with an increasing extent of mouth breathing. To our knowledge, this is the first study to evaluate the impact of breathing patterns on the prevalence of atopic dermatitis. Although two studies report that mouth-breathing children with positive skin-prick test results have higher prevalences of asthma and sleep apnea than those with negative results [9, 33], they did not compare mouth breathers with nasal breathers.

No previous study has analyzed the prevalence of mouth breathing among Japanese children. In the present study, the prevalences of MBD and MBS were 35.5% and 45.9%, respectively; however, these results were based on a questionnaire that has not been assessed for validity. The studies from other countries report a wide range of the prevalence of mouth breathing, 4–56% [1–8]. Brazil has the highest prevalence, which exceeds 50% based on clinical assessment [1–3]. On the other hand, the lowest prevalence is in India, at 4–7% based on clinical assessment [6–8]. The prevalences of mouth breathing in England and New Zealand are 23% and 19%, respectively [4, 5]. However, direct comparison among studies is difficult owing to varying criteria. Standardized criteria for mouth breathing using clinical assessment and questionnaires are required to more precisely investigate differences in mouth breathing prevalence.

Atopic dermatitis is a chronic skin disorder characterized by pruritus and inflammation that mostly develops during childhood and is strongly associated with the allergic history of patients and relatives [34]. Filaggrin, which is encoded by the *FLG* gene, is a crucial protein for skin barrier function [35, 36]. Two meta-analyses show that children with an abnormal *FLG* gene are 3.12–4.78 times more likely to have atopic dermatitis than normal subjects [37, 38]. On the other hand, the prevalences of chronic pediatric diseases, including atopic dermatitis as well as asthma and allergic rhinitis, vary greatly worldwide [27]. The prevalences of these diseases have increased in the late 20^th^ century [39–41], although this trend has not held true in the last 10 years [39, 42]. These findings suggest that, in addition to genetic factors, environmental factors play important roles in these diseases.

This was a population-based cross-sectional study; therefore, causal relationships cannot be determined. However, if mouth breathing is shown to contribute to atopic dermatitis in future cohort studies, guidance to avoid mouth breathing should be provided to children and parents/guardians to prevent atopic dermatitis. Furthermore, otolaryngologist help should be considered, as necessary, if children find it difficult to quit mouth breathing.

It is possible that periodontal disease and/or tonsillitis might mediate the mechanism underlying any association between mouth breathing, as an environmental factor, and atopic dermatitis [18–26]. Satoh et al. suggested that immune reactions mediated by bacterial–immune complexes, superantigens, or toll-like receptors might induce skin diseases [20]. However, the present questionnaire did not collect periodontal information. Furthermore, although the present results showed no significant relationship between history of tonsillitis and the prevalence of atopic dermatitis, inquiring about the number of repeated tonsillitis episodes might have revealed an association. On the other hand, it is possible that children could have mild nasal congestion unnoticed by their guardians. Thus, case–control studies with otolaryngological diagnostic tests are required to confirm the relationship between mouth breathing and atopic dermatitis. Furthermore, sleep disturbance due to the intense pruritus of atopic dermatitis [43] can cause daytime sleepiness [44], which might in turn cause behavioral mouth breathing.

According to the Japanese Ministry of Health, Labour, and Welfare, the prevalences of atopic dermatitis in 3- and 6–7-year-old children are 13.2% and 11.8%, respectively [45]. Meanwhile, the prevalence of asthma in a study of 34,699 children aged 4–5 years at randomly selected nurseries in Japan was 11.2%, and the lifetime prevalences of atopic dermatitis, asthma, and allergic rhinitis were 16.0%, 16.1%, and 17.6%, respectively [46]. In the present study, the prevalences of atopic dermatitis, asthma, and allergic rhinitis were 12.6%, 9.8%, and 13.0%, respectively; the lifetime prevalences were 15.4%, 14.5%, and 18.2%, respectively. Thus, the present results are similar to those of previous studies, suggesting the subject group is representative of Japanese children aged 2–6 years.

In this study, 45.9% of children had family members who smoked. This is similar to the prevalence of smokers among Japanese men and women 20–40 years old: 40% and 12%, respectively [47]. However, having a family member who smoked was not associated with disease prevalence. Possible reasons for this are the low rate of smokers who smoke around their children (7.1%) and parents who stop smoking when their child develops a disease.

There are some limitations in this study, as already mentioned. First, the diagnoses of atopic dermatitis and mouth breathing were dependent on the questionnaire results. Because we created the questions and criteria for mouth breathing, the lack of experimental data and questionnaire validity limits the strength of our findings. Second, the valid response rate was comparatively low at 45.2%. Third, as this was a cross-sectional study, causal relationships cannot be determined. Although we cannot exclude facial injuries affecting nasal breathing, such cases would be rare and are unlikely to affect the results. The effects of common cold and flu appear quite small, because we inquired about the normal state of the children in the questionnaire. Finally, regarding genetic factors, only the parents’ information was collected and not that of sibling or grandparents. Therefore, genetic factors may have been underestimated.

## Conclusion

Mouth breathing is significantly associated with atopic dermatitis in Japanese preschool children aged 2–6 years. Additional case–control and cohort studies are required to confirm this relationship. Furthermore, studies targeting school children and adults would also help clarify this association.

## Supporting Information

S1 Appendix Association between mouth breathing and disease prevalence (full data). (DOCX)
