# Supplementary material for: Association between Mouth Breathing and Atopic Dermatitis in Japanese Children 2–6 years Old: A Population-Based Cross-Sectional Study
Source: PLoS One. 2015 Apr 27;10(4):e0125916. doi: 10.1371/journal.pone.0125916 (PMC4411141; doi:10.1371/journal.pone.0125916)
Supplement: S1 Appendix — (DOCX) [file pone.0125916.s001.docx]

**S1 Appendix. Association between mouth breathing and disease prevalence (full data)**

|  |  | Total | Atopic dermatitis | | |  | Asthma | | |  | Allergic rhinitis | | |  |
| --- | --- | --- | --- | --- | --- | --- | --- | --- | --- | --- | --- | --- | --- | --- |
|  |  |  | - | + |  |  | - | + |  |  | - | + |  |  |
|  |  | *n* = 468  (%) | *n* = 409  (%) | *n* = 59  (%) | OR | 95% CI | *n* = 422  (%) | *n* = 46  (%) | OR | 95% CI | *n* = 407  (%) | *n* = 61  (%) | OR | 95% CI |
| Breathes with mouth ordinarily | Positive | 158 | 130 | 28 | 1.94 | 1.12 –3.37 | 136 | 22 | 1.93 | 1.04–3.56 | 126 | 32 | 2.46 | 1.43–4.24 |
|  |  | (33.8) | (82.3) | (17.7) |  | (*p* = 0.017) | (86.1) | (13.9) |  | (*p* = 0.034) | (79.7) | (20.3) |  | (*p* < 0.001) |
|  | Negative | 310 | 279 | 31 |  |  | 286 | 24 |  |  | 281 | 29 |  |  |
|  |  | (66.2) | (90.0) | (10.0) |  |  | (92.3) | (7.7) |  |  | (90.6) | (9.4) |  |  |
| Mouth is open ordinarily | Positive | 218 | 176 | 42 | 3.27 | 1.80–5.94 | 192 | 26 | 1.56 | 0.84–2.88 | 182 | 36 | 1.78 | 1.03–3.07 |
|  |  | (46.6) | (80.7) | (19.3) |  | (*p* < 0.001) | (88.1) | (11.9) |  |  | (83.5) | (16.5) |  | (*p* = 0.037) |
|  | Negative | 250 | 233 | 17 |  |  | 230 | 20 |  |  | 225 | 25 |  |  |
|  |  | (53.4) | (93.2) | (6.8) |  |  | (92.0) | (8.0) |  |  | (90.0) | (10.0) |  |  |
| Mouth is open when chewing | Positive | 159 | 131 | 28 | 1.92 | 1.10–3.33 | 135 | 24 | 2.32 | 1.26–4.28 | 134 | 25 | 1.41 | 0.82–2.45 |
|  |  | (34.0) | (82.4) | (17.6) |  | (*p* = 0.019) | (84.9) | (15.1) |  | (*p* = 0.006) | (84.3) | (15.7) |  |  |
|  | Negative | 309 | 278 | 31 |  |  | 287 | 22 |  |  | 273 | 36 |  |  |
|  |  | (66.0) | (90.0) | (10.0) |  |  | (92.9) | (7.1) |  |  | (88.3) | (11.7) |  |  |
| **MBD** | Positive | 166 | 134 | 32 | 2.43 | 1.40–4.23 | 142 | 24 | 2.15 | 1.17–3.97 | 137 | 29 | 1.79 | 1.04–3.07 |
|  |  | (35.5) | (80.7) | (19.3) |  | (*p* = 0.001) | (85.5) | (14.5) |  | (*p* = 0.013) | (82.5) | (17.5) |  | (*p* = 0.035) |
|  | Negative | 302 | 275 | 27 |  |  | 280 | 22 |  |  | 270 | 32 |  |  |
|  |  | (64.5) | (91.1) | (8.9) |  |  | (92.7) | (7.3) |  |  | (89.4) | (10.6) |  |  |
|  |  |  |  |  |  |  |  |  |  |  |  |  |  |  |
| Snoring | Positive | 136 | 110 | 26 | 2.14 | 1.23–3.74 | 119 | 17 | 1.49 | 0.79–2.82 | 99 | 37 | 4.80 | 2.74–8.41 |
|  |  | (29.1) | (80.9) | (19.1) |  | (*p* = 0.007) | (87.5) | (12.5) |  |  | (72.8) | (27.2) |  | (*p* < 0.001) |
|  | Negative | 332 | 299 | 33 |  |  | 303 | 29 |  |  | 308 | 24 |  |  |
|  |  | (70.9) | (90.1) | (9.9) |  |  | (91.3) | (8.7) |  |  | (92.8) | (7.2) |  |  |
| Mouth is open during sleeping | Positive | 234 | 195 | 39 | 2.14 | 1.21–3.80 | 210 | 24 | 1.10 | 0.60–2.03 | 194 | 40 | 2.09 | 1.19–3.67 |
|  |  | (50.0) | (83.3) | (16.7) |  | (*p* = 0.008) | (89.7) | (10.3) |  |  | (82.9) | (17.1) |  | (*p* = 0.009) |
|  | Negative | 234 | 214 | 20 |  |  | 212 | 22 |  |  | 213 | 21 |  |  |
|  |  | (50.0) | (91.5) | (8.5) |  |  | (90.6) | (9.4) |  |  | (91.0) | (9.0) |  |  |
| Mouth is dry when your child gets up | Positive | 295 | 253 | 42 | 1.52 | 0.84–2.77 | 262 | 33 | 1.55 | 0.79–3.03 | 255 | 40 | 1.14 | 0.65–2.00 |
|  |  | (63.0) | (85.8) | (14.2) |  |  | (88.8) | (11.2) |  |  | (86.4) | (13.6) |  |  |
|  | Negative | 173 | 156 | 17 |  |  | 160 | 13 |  |  | 152 | 21 |  |  |
|  |  | (37.0) | (90.2) | (9.8) |  |  | (92.5) | (7.5) |  |  | (87.9) | (12.1) |  |  |
| **MBS** | Positive | 215 | 177 | 38 | 2.37 | 1.34–4.18 | 192 | 23 | 1.20 | 0.65–2.20 | 177 | 38 | 2.15 | 1.23–3.73 |
|  |  | (45.9) | (82.3) | (17.7) |  | (*p* = 0.002) | (89.3) | (10.7) |  |  | (82.3) | (17.7) |  | (*p* = 0.006) |
|  | Negative | 253 | 232 | 21 |  |  | 230 | 23 |  |  | 230 | 23 |  |  |
|  |  | (54.1) | (91.7) | (8.3) |  |  | (90.9) | (9.1) |  |  | (90.9) | (9.1) |  |  |
|  |  |  |  |  |  |  |  |  |  |  |  |  |  |  |
| Nasal congestion | Positive | 97 | 79 | 18 | 1.83 | 1.00–3.36 | 79 | 18 | 2.79 | 1.47–5.30 | 61 | 36 | 8.17 | 4.58–14.56 |
|  |  | (20.7) | (81.4) | (18.6) |  | (*p* = 0.047) | (81.4) | (18.6) |  | (*p* = 0.001) | (62.9) | (37.1) |  | (*p* < 0.001) |
|  | Negative | 371 | 330 | 41 |  |  | 343 | 28 |  |  | 346 | 25 |  |  |
|  |  | (79.3) | (88.9) | (11.1) |  |  | (92.5) | (7.5) |  |  | (93.3) | (6.7) |  |  |

OR: odds ratio, 95% CI: 95% confidence interval, MBD: mouth breather in daytime, MBS: mouth breather during sleep.
